# Class, gender and the work of working‐class women amid turbulent times

**DOI:** 10.1111/1468-4446.13147

**Published:** 2024-09-19

**Authors:** Tracey Warren, Luis Torres, Clare Lyonette, Ruth Tarlo

**Affiliations:** ^1^ University of Nottingham Nottingham UK; ^2^ Universidad del Desarrollo Santiago Chile; ^3^ University of Warwick Coventry UK

**Keywords:** Covid‐19, employment, pandemic, risks, wages, working‐class women

## Abstract

The article focuses on the work of working‐class women (WCW) amid turbulent times. Its timespan is just prior to and during the Covid‐19 pandemic in the UK. The women's work, and the key skills involved, are fundamental to everyday lives, but both have been under‐valued and under‐rewarded. The pandemic shone a fresh light on the societal importance of this work and highlighted how its under‐valuation and the women's systemic low pay and inferior working conditions have serious ramifications not only for individual workers and their families but for the provision of key services. The article centres WCW, at the intersection of classed and gendered disadvantage, to ask about inequalities in work experiences. Analysing nationally representative samples of thousands of workers in the UK prior to and as Covid‐19 rolled out, we compare WCW with other workers. We show that the women faced both persistent and new inequalities at work: enduring low earnings, pandemic‐led risks to jobs and paid hours, little opportunity to work from home or flexibly, and stressful key working roles. We reveal the heavily classed nature of some of these findings, show that others were more strongly gendered, while still others were classed and gendered outcomes that require intersectional analyses of the women's working lives.

## 1 INTRODUCTION

The article explores the paid work of working‐class women (WCW) just prior to and during the Covid‐19 pandemic. It centres WCW because they are living at the intersection of both classed and gendered disadvantages (Armstrong, 2006; Bradley, 2020; Friedman, 2022; Hebson, 2009; Warren, 2000) and they are engaged extensively in forms of employment that are core to societal functioning. They are frequently found working in the so‐called ‘5C’ jobs of caring, cleaning, catering, clerical work, and cashiering: cleaning buildings; preparing and serving food; administering institutions; staffing shops and, providing frontline care for children, sick and frail elderly (Perrons, 2009). Though the work that they do, and the skills involved, are fundamental to everyday lives, both have long been under‐valued and under‐rewarded. This under‐valuation has ramifications that include but go beyond negative consequences for individual workers and their families. Systemic low pay and inferior working conditions have produced labour shortages in key services, low staff morale and high turnover, and inadequate provision (Eurofound, 2023; ILO, 2023). The arrival of the Covid‐19 pandemic highlighted the importance of WCW's work and challenged its under‐valuing via raising serious questions about what roles and which workers are essential or key (Farris & Bergfeld, 2022; Farris et al., 2024; Remery et al., 2022).

The pandemic context signalled too the importance of renewed sociological attention to WCW's work amid turbulent times. The article was inspired by 1980s classic UK sociology when, amid deep economic turbulence, there was direct attention to class, gender and WCW's working lives (e.g., Bradley, 1989; Cavendish, 1982; Crompton & Jones, 1984; Elson & Pearson, 1980; Pollert, 1981; Westwood, 1985). Contemporary sociological attention to the work of WCW has been more scattered since: insights into the women's employment are similarly found in studies of work that directly employ class (e.g., Armstrong, 2006; Crompton & Lyonette, 2008; Hebson, 2009; Snee & Goswami, 2021; Warren, 2000; Warren & Lyonette, 2018) but also across class‐based research where employment is not the primary focus (e.g., Johansson & Jones, 2019; Lawler, 2005; McCarthy et al., 2000; Parsons et al., 2024; Shildrick et al., 2012; Skeggs, 1997) and employment‐based research that does not utilise a class lens (e.g., McMunn et al., 2020; Nightingale, 2019; Patrick et al., 2022).

This article draws together and builds upon extant knowledge of WCW's work to add new insights into their experiences amid deep turbulence. Our temporal focus is during and just prior to Covid‐19. The pandemic has triggered a substantial literature and, for our interest in paid work, many studies show the unequal risks it brought. We know that employed women and men experienced the pandemic very unevenly due to their different combinations of paid and unpaid work roles and responsibilities (ILO, 2023; Petts et al., 2023; Zhou & Kan, 2021) but classed analyses of work inequalities during the pandemic have been more muted (McKenzie, 2021) and research examining class, gender and work rarer still (Chatot et al., 2023). In this article, we deploy a classed and gendered lens and compare the WCW's experiences with other workers, focussing on inequalities in jobs and finances and access to the new protections put in place to help workers in the face of the extraordinary and shifting burdens of the pandemic.

## 2 SOCIOLOGY AND THE WORK OF WORKING‐CLASS WOMEN

Class inequalities strongly shape the sociological understanding of working lives. Savage (1999) discusses a golden age of male‐dominated work sociology (1955–1975) when the work experiences of working‐class men (WCM) were central in influential studies and to the formation of class‐based theories of work (Eidlin, 2016). This was followed by a period of deep recession in the 1980s that saw sociological interest in capturing the unequal ways that class and gender together (Roth & Dashper, 2016) intensify or minimise the impacts of economic turbulence on work and workers. Feminist sociologists usefully centred WCW's experiences because of their classed *and* gendered disadvantaged positioning (Hebson, 2009). Amid recessionary instability, research in the UK by Bradley (1989), Crompton (e.g., Crompton & Jones, 1984), Elson (e.g., Elson & Pearson, 1980), Glucksmann (as Cavendish, 1982), Pollert (1981) and Westwood (1985), alongside for example, Milkman (1982) in the USA, all made the case that much of WCW's work is crucial for the economy and for the well‐being of women, their families and communities, but it is routinely poorly rewarded and undervalued. In focussing on WCW and their work, these studies made clear that the unequal ramifications of the 1980s recession included but went beyond a loss of jobs that, in the UK, largely affected men (Rubery & Tarling, 1988). They showed increased precariousness among those still employed and a heavy burden of unpaid domestic work falling on WCW especially as they cared for families and managed households while experiencing or fearing financial hardship (and see Pahl & Wallace, 1985).

Focussed attention on WCW within the sociology of employment grew more fragmented after the 1980s. As Milkman et al. (2021) discuss, class and gender together have often not framed sociological analyses of working lives. In the UK, while studies do focus directly on WCW's employment *and* use class and gender to do so (e.g., Armstrong, 2006; Crompton & Lyonette, 2008; Hebson, 2009; Smith & McBride, 2021; Snee & Goswami, 2021; Vagni, 2020; Walters, 2005; Warren, 2000, 2016; Warren & Lyonette, 2018, 2020), many insights into the women's working lives come either from outside the sociology of employment or from non‐classed studies. We know about the characteristics of the women's paid work from class‐focused research that focuses primarily on poverty (e.g., Parsons et al., 2024; Shildrick et al., 2012), classed communities (e.g., McKenzie, 2015) and families and identity (e.g., Johansson & Jones, 2019; Lawler, 2005; McCarthy et al., 2000; Skeggs, 1997). There are also valuable insights from employment‐based research into women workers with low‐incomes or lower levels of formal qualifications (e.g., Kowalewska, 2023; McMunn et al., 2020; Nightingale, 2019; Patrick et al., 2022). Combined, these sources show that women and men work in highly segregated occupations, but WCW, at the intersections (Crenshaw, 1988) of class and gender disadvantage, are even more narrowly clustered than are other workers. In the UK, they are over‐concentrated in low paid, often part‐time, often lower quality jobs, especially when they have dependent children and/or other caring responsibilities, undermining their prospects and increasing their vulnerability to hardship.

For women in general, what many do in their paid work has strong parallels with the work they do, unpaid, in the home, and so women's abilities have been viewed as natural or innate: no (apparent) skills means no recognition in the form of wages or social value. For WCW, many of their jobs call upon especially stereotypical constructions of supposedly natural feminine aptitudes (Skeggs, 1997). Even after controlling for recognised and objective measures of skill, WCW have been seen, and self‐identify too, as less than their skills (Friedman, 2022; Horrell et al., 1990; Skeggs, 1997). The misrecognition and disregard of their skills talks strongly to how WCW are valued in society, a very powerful theme in recent decades of sociology in which class and gender are brought together (Hebson, 2009; Johansson & Jones, 2019; Lawler, 2005; McKenzie, 2015; Mellor et al., 2014; Skeggs, 1997), reinvigorating sociology in many ways.

Building upon the 1980s sociological centring of WCW's lives amid recessionary turmoil, this article focuses on the women's paid work experiences amid pandemic turbulence. Covid‐19 is notable for creating a new urgency around defining whose labour is essential and whose work is valued (Eurofound, 2023; ILO, 2023). It made the paid work of WCW more visible and motivated our aim in this article for its sociological recentring.

## 3 RISKS TO AND PROTECTIONS OF PAID WORK DURING THE PANDEMIC

The pandemic rapidly brought unequal risks to jobs, pay levels, and opportunities for flexible and home‐working (Blundell et al., 2022; Hudde et al., 2023; ILO, 2023; Patrick et al., 2022; Robinson et al., 2021; Wood & Bennett, 2023). These risks were firmly gendered: women and men experienced the pandemic very unevenly due to their different combinations of paid and unpaid work roles and responsibilities (ILO, 2023; Petts et al., 2023; Zhou & Kan, 2021). Compared with the considerable attention paid to gender and work, classed analyses of work inequalities amid pandemic pressures were more muted, as Chatot et al. (2023) discuss. Over‐concentrated in jobs that were strongly at risk (such as lower waged jobs in retail and services), and with their finances under threat (Herman et al., 2021; Raphael & Schneider, 2023; Robinson et al., 2021; Wu, 2023; Zhou & Kan, 2021), working‐class experiences were nevertheless side‐lined in debate and analyses, certainly in the early days of Covid‐19 (McKenzie, 2021).

In the face of risks to jobs and finances, new pandemic protections were put in place by governments and firms. A major component of initiatives was a forced expansion in home‐working and more widespread availability of flexible working, but there were deep inequalities in access to both (Chung et al., 2021; McPhail et al., 2023). A linked innovation was the official categorisation of workers as key or non‐key to designate who was still allowed to go out to work during lockdowns. Positively, on the one hand, this strategy stimulated valuable debate over whether the lack of recognition accorded to many workers was under challenge (Beck et al., 2020; Eurofound, 2023; ILO, 2023; Remery et al., 2022). This development is especially relevant for those working in lower paid jobs previously dismissed as low‐value and low skilled, often undertaken by WCW as we discuss. Covid‐19 certainly created opportunities for shows of appreciation for key workers: carers and health workers were applauded in many countries (Farris & Bergfeld, 2022; Lloyd et al., 2024; Wood & Skeggs, 2020). On the other hand, being deemed key meant that many workers had to leave their homes for work, facing unsafe work conditions and heightening their exposure to the virus, hence a heroic worker rhetoric (Hales & Tyler, 2022).

The third protection to mitigate both job and financial risk among workers in the UK was the ‘Coronavirus Job Retention Scheme’. Known commonly as the furlough scheme, it allowed for workers to go on a temporary full or partial leave, paid at 80% of their wages (capped at £2500 a month, unless otherwise agreed). It closed at the end of September 2021. Furlough gave workers some job and financial protection in comparison to non‐furloughed workers, but lower paid workers were less likely to have their pay topped up than were higher earners on furlough (Adams‐Prassl, Balgova, & Qian, 2020).

Covid‐19 brought diverse risks to workers and unequal coverage by pandemic protections, but highlighted the important work of WCW and signalled that its under‐valuation has real societal ramifications. We explore class and gender inequalities at work in the UK amid pandemic turmoil via centring the experiences of WCW.

## 4 RESEARCH METHODS

The article draws upon the secondary analysis of two datasets. Using a single data source can produce errors due to for example, non‐response and question wording (Firebaugh, 2018), and so we use two complementary high‐quality nationally representative datasets. The first is the Labour Force Survey (LFS) that provides the official UK measures of employment and unemployment for the entire population. We draw overall population trends based on the 2019/20/21 releases. Respondents are interviewed for five successive waves at three‐month intervals. Four quarters releases are supported in a typical year: Jan‐Mar, Apr‐Jun, Jul‐Sep, Oct‐Dec. In 2020 and 2021, additional non‐calendar quarter data were released in response to the context of the pandemic (ONS, 2021). In addition to the four calendar quarters per year, 2020 included another eight releases of data. We tracked trends before and during the pandemic, considering the March/May 2020 quarter as the reference point. We also traced pre‐pandemic trends back to the first quarter of 2019. We exclude November 2020 to January 2021 and December 2020 to February 2021 due to changes in the occupational classification coding scheme.

The second source is the UK Household Longitudinal Study (UKHLS) that samples around 100,000 individuals across 40,000 households. We draw on data from the pre‐pandemic full wave plus the Covid‐19 study, a monthly survey of the experiences and reactions of the UK population to the pandemic (University of Essex, 2022). A questionnaire entered the field in April 2020 and was carried out at roughly monthly intervals for 12 months, plus a final wave in September 2021, with questions adapted as the pandemic situation changed. All UKHLS sample members aged 16+ and who had taken part in one of the two previous waves of the main study were invited to participate: 17,450 completed the first Covid survey.

Our analyses consider the economically active working age population (18–65 years old), while excluding self‐employed workers whose worktime, systems of remuneration, and levels of autonomy vary substantially from employees, and who experienced different covid protections. The characteristics of both datasets are summarised in Supporting Information S1: Appendix 1.

### 4.1 Variables

Class is our key variable, and our approach is to use occupational class. Shaping this decision was Crompton's (2010) influential argument that class and employment are firmly connected. As she puts it, class outcomes for most workers are strongly related to the kinds of occupations available to them. Occupational class brings challenges in classifying those outside paid work; occupation does not tell us about class of origin (Friedman, 2022; Friedman & Laurison, 2019; Snee & Goswami, 2021) or capitals (though occupation and capitals are linked. Savage et al., 2014), and an occupation might not match classed self‐identity (Crew, 2021). This article uses occupational class specifically to analyse the working conditions of employees. It ‘does not furnish a complete understanding of the complexities of class’, but it is a useful and commonly used proxy in sociology (Crompton, 2010, p. 22).

We use the National Statistics Socio‐economic Classification’ (‘NS‐SEC’. ONS, 2022). NS‐SEC is one of the most widely used occupational class measures (Vagni, 2020). It was devised by Rose et al. (2005) to provide a sociologically informed official socio‐economic classification, building upon Erikson and Goldthorpe's (1992) Weberian‐rooted class schemas that distinguish between employees in higher‐level ‘service’ jobs, who have more discretion over their work, from those in intermediate roles and those with a ‘labour contract’ that pays for a quantity of time. We examine ‘Semi‐routine’ (e.g., care‐workers, retail assistants) and ‘Routine’ workers (e.g., cleaners, waiting staff, bar staff) as working‐class workers: their contracts involve direct exchanges of money and effort. As the sample characteristics show, the two largest groups of workers were in managerial/professional and routine‐/semi‐routine occupations. Women in lower supervisory/technical jobs had work experiences in some ways similar and sometimes worse to those we categorise as working‐class but they only account for a maximum 4%–5% of the female employee samples.

Our other key variables include age; sex; employment status (to identify employment levels and then select employees); key worker status; working from home or not; flexible work arrangements; weekly hours in paid work; weekly take‐home earnings; whether people are making savings and whether they are managing financially (detailed in Supporting Information S1: Appendix 2).

### 4.2 Analytical strategy

The LFS and UKHLS are analysed together to expose and track overarching patterns in working lives to see how WCW fared. Drawing on Savage (2024) in this journal, who contends that descriptive analyses are highly valuable in sociology because they reveal key associations and patterns, potentially opening up new worlds to audiences and disclosing new possibilities for research, trend analyses were implemented on the LFS supported by descriptive analyses of UKHLS and logistic regressions. The purpose of the regressions is primarily to see whether the significance of the key explanatory variables (class and gender) persists or disappears after controls are added, rather than the extent to which the models explain variance in the dependent variables (the Nagelkerke R square). Odds ratios (and 95% confidence intervals) are provided to show how WCW fare relative to a reference group of non WCM. In the descriptive analysis, for each indicator, we show how WCW fare compared with workers in the three other classed/gendered categories. All analyses were implemented using the recommended, provided population weights to allow for sample bias correction including differential non‐responses across waves (ONS, 2021; University of Essex, 2022).

## 5 FINDINGS

### 5.1 Pandemic risks to jobs and finances

We look at WCW's paid work via an examination of their risks of job loss, of being in highly precarious employment, and whether they worked no hours despite being employed, when compared with other workers. Their financial situations are explored via their earnings.

#### 5.1.1 Jobs

**FIGURE 1:**
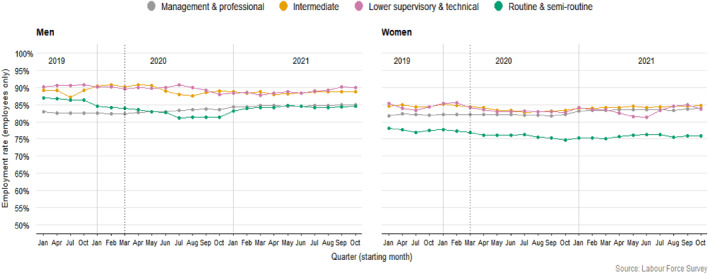
Employment rates over time (weighted data).

Entering the pandemic, many WCW were over‐concentrated in jobs likely to be heavily impacted but in diverse ways, whether because they were in soon to be shut‐down sectors or were deemed key. The LFS analysis reveals stark variation in employment levels shaped by class and gender. In the UK, men's rates of employment generally fall the most in periods of economic crisis (Rubery & Rafferty, 2013), and our analysis shows that the Covid‐19 pandemic was no different, but it was a firmly classed picture. WCM saw the steepest falls in their employment (Figure 1), fuelled by job loss among assemblers, machine operators, porters and messengers, and plumbers. Yet we can see that WCW were not spared (with those in cleaning, shop work and care work most impacted). In general, however, because women were up to two times more likely to be employed in public sector roles (particularly health, education, and local government), their employment levels were better protected.

**FIGURE 2:**
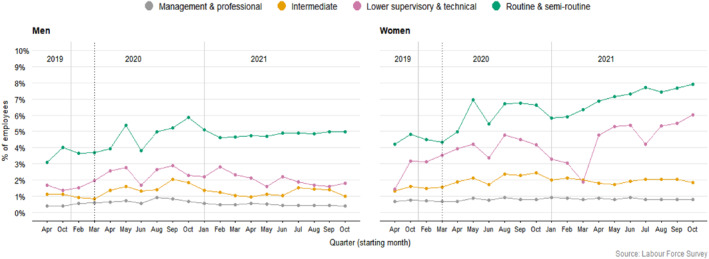
Evolution of zero hours contracts (weighted data).

Job loss is not the only consequence of economic turbulence, as discussed. For those still in employment, precarious contractual arrangements increased with Covid‐19. For example, Figure 2 shows a growing percentage of employees with the very precarious zero‐hour contracts, increasing most for WCW.

**FIGURE 3:**
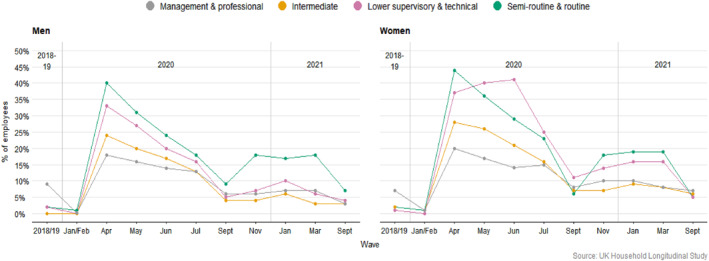
Employed but doing 0 hours of work (weighted data).

One notable new phenomenon with the pandemic, that clearly impacted WCW heavily, was the large and rapid increase in the numbers of the still‐employed who saw their hours in paid work cut to zero. The UKHLS show the percentages shooting up to 44% of WCW, and 40% for WCM, by April 2020 (Figure 3). Using data from the major lockdown in January 2021 too, binary logistic regressions identify which employees, when asked how many hours they worked last week, reported zero. The regressions confirm and quantify that the no‐hours‐employed phenomenon was a more strongly classed than gendered pandemic experience (summary Table 1). We examined the class/gender categories while adding in controls for baseline (January/February 2020) hours worked and wages and current keyworker status (model 2), and then age, ethnicity, couple and parental status (model 3). When compared with a reference group of non‐working‐class men (workers in the least disadvantaged of intersecting class/gender categories), WCW stood out with significant odds ratios in the full Models 3 (WCW: 3.5 and 2.4 for WCM in April and January, *p* < 0.01).

**TABLE 1:** Employed but working no hours during major lockdowns.

	April 2020						January 2021					
	Model 1		Model 2		Model 3		Model 1		Model 2		Model 3	
	Odds ratio	95% CI	Odds ratio	95% CI	Odds ratio	95% CI	Odds ratio	95% CI	Odds ratio	95% CI	Odds ratio	95% CI
Class and gender^a^												
Women ‐ working class	2.8***	(2.5, 3.3)	3.6***	(3.0, 4.4)	3.5***	(2.8, 4.3)	2.9***	(2.3, 3.7)	2.8***	(2.0, 3.9)	2.4***	(1.7, 3.5)
Women ‐ non working class	1.1	(1.0, 1.3)	1.2*	(1.1, 1.4)	1.2**	(1.1, 1.4)	1.3**	(1.1, 1.7)	1.4**	(1.1, 1.9)	1.4**	(1.1, 1.8)
Men ‐ working class	2.4***	(2.1, 2.9)	3.4***	(2.8, 4.1)	3.3***	(2.7, 4.0)	2.5***	(1.9, 3.3)	2.4***	(1.7, 3.4)	2.4***	(1.7, 3.4)
Constant	0.3***		0.5***		0.6***		0.1***		0.2***		0.2***	
Nagelkerke R square	0.05		0.3		0.27		0.04		0.14		0.14	
N	7542		6853		6781		4919		4208		4193	

*Note*: Summary binary logistic regression (full) models. Model 2 controls for hours worked and low wages (earning less than two‐thirds of the median net weekly wage in the sample that month) in Jan/Feb 2020, and current keyworker status. Model 3 then adds in controls for age, ethnicity, couple and parental status. Employed women and men aged 18–65, excluding the self‐employed. Employed in month of focus and Wave j.

***p < 0.01, **p < 0.05, *p < 0.1.

*Source*: UKHLS weighted data.

^a^ Reference: Men non‐working class.

#### 5.1.2 Finances

**FIGURE 4:**
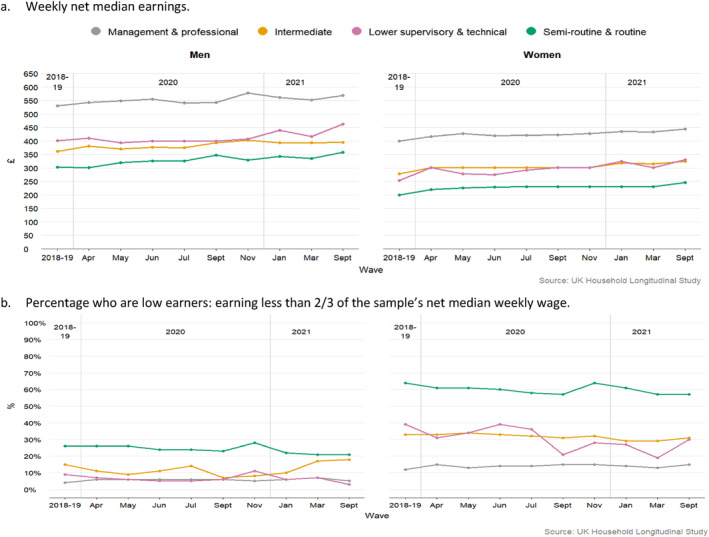
Financial situations over time (weighted data).

We next ask how WCW were faring financially. It is notable both just how poorly they were already doing in their waged income before the disruptions of Covid‐19, and that their disadvantaged positions persisted through the shifting pandemic months. For example, while WCW were earning just over £200 (net) a week on average each wave, managerial/professional men were consistently earning more than twice that amount (Figure 4A). Figure 4B displays how, relative to other workers, WCW were by far the group most likely to be very low earners: on less than 2/3 of the net median weekly wage for all workers in the sample that month, again quite stable across time. Logistic regressions affirm that WCW remained the group of workers most likely to be low waged each wave, after controls were added (odds ratios of 9.1 in 2018‐19, 16.0 in April 2020, 19.3 in November 2020, *p* < 0.01. Table 2).

**TABLE 2:** Summary binary logistic regression (full) models for employees with low wages.

	Low waged^b^					
	2018‐19		April 2020		November 2020	
	Odds ratio	95% CI	Odds ratio	95% CI	Odds ratio	95% CI
Class and gender^a^						
Women ‐ working class	9.1***	(7.6, 10.9)	16.0***	(12.5, 20.7)	19.3***	(14.1, 26.5)
Women ‐ non working class	1.4***	(1.2, 1.6)	2.9***	(2.3, 3.7)	2.8***	(2.2, 3.7)
Men ‐ working class	3.3***	(2.7, 4.0)	3.2***	(2.5, 4.3)	3.7***	(2.6, 5.2)
Constant	0.06***		0.02***		0.03***	
Nagelkerke R square	0.56		0.48		0.53	
N	13,698		6393		4330	

*Note*: Employed women and men aged 18–65, excluding the self‐employed. Employed in month of focus and Wave j.

***p < 0.01, **p < 0.05, *p < 0.1.

*Source*: UKHLS weighted data.

^a^ Reference: Men non‐working class.

^b^ Low waged = earning less than two‐thirds of the median net weekly wage in the sample that month. Controls include current hours worked, keyworker status (April only), furlough (2020 only), age, ethnicity, couple, parental status.

### 5.2 Pandemic protections

We next explore how WCW fared in coverage by three major pandemic protections.

#### 5.2.1 Working from home

Pre‐pandemic, having some autonomy over where you carry out your work was associated with improved work‐life balance and well‐being, especially when working from home also meant a comfortable and suitable work environment (Felstead, 2022). Even when work tasks can technically and even easily be done from home for some or all the time (various types of semi‐routine office work for example), however, the flexibility to work from home has not been equally available to all groups of eligible workers (Hassard & Morris, 2024). With the onset of the pandemic, many more workers had to work from their homes, providing a ‘sea change’ in working conditions for many (Fan & Moen, 2022; ILO, 2023; McPhail et al., 2023; Raphael & Schneider, 2023).

**FIGURE 5:**
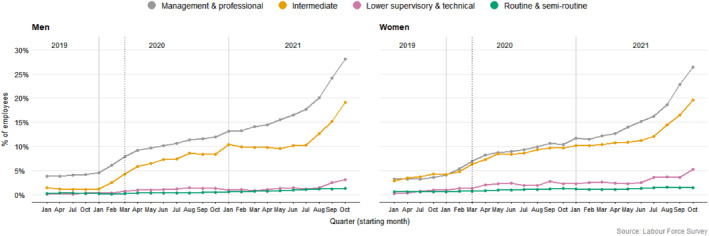
Percentage of people working from home over time (weighted data).

Our analysis of the LFS in Figure 5 demonstrates just how rapidly working from home increased from the first lockdown and shows clearly that this was a strongly classed phenomenon. Working‐class women and WCM were little likely to be afforded this form of protection. Using the UKHLS too, logistic regressions affirmed the significance of class (Table 3).

**TABLE 3:** Summary binary logistic regression (full) models for working at home (WAH) and flexible work arrangements (FWAs).

	WAH^b^ or not		FWAs^c^ or not	
	Odds ratios	95% CI	Odds ratios	95% CI
Class and gender^a^				
Women ‐ working class	0.4***	(0.3, 0.6)	0.6***	(0.5, 0.8)
Women ‐ non working class	1.5***	(1.3, 1.8)	1.7***	(1.5, 2.0)
Men ‐ working class	0.1***	(0.1, 0,1)	0.2***	(0.1, 0.3)
Constant	3.5***		0.7***	
Pseudo *R* ^2^	0.46		0.14	
N	5317		4216	

*Note*: Employed women and men aged 18–65, excluding the self‐employed. Employed in month of focus and Wave j.

***p < 0.01, **p < 0.05, *p < 0.1.

*Source*: UKHLS weighted data.

^a^ Reference: Men non‐working class.

^b^ WAH: always/often worked at home in April 2020. Controls include current hours worked, keyworker status, age, ethnicity, couple status, parental status.

^c^ FWA: one FWA or more available at the workplace in June 2020. Controls include current hours worked, keyworker status, wages, age, ethnicity, couple status, parental status.

We also explored who had access in their workplace to a range of flexible working arrangements (FWAs). Good quality FWAs are known to facilitate balancing a job with the demands of care and housework, with those pressures intensifying during the pandemic, for women especially. There is mixed evidence however, on how class and gender together shaped flexibility amid economic uncertainty (Damaske, 2020). Access to FWAs during covid was, as it had been before, firmly classed (see Table 3. Also Brega et al., 2023) and, because women workers reported access to lower‐quality FWAs than did men overall, WCW stood out with ‘double disadvantage’ (Friedman, 2022). Lower‐quality FWAs are driven by employers' desire to reduce labour cost, rather than providing access to working conditions that facilitate balancing work and home responsibilities (Adams‐Prassl, Balgova, & Qian, 2020).

#### 5.2.2 Furloughing

The new no‐paid hours experience among the still employed, reported earlier, was driven in part by furlough arrangements. The UHKLS provides a variable that tallies up whether participants had been furloughed in any previous survey month. Responses in November 2020, the last time that this cumulative variable is available, showed how strongly classed furloughing was, impacting WCM especially (Table 4: significant odds ratios for WCM at 2.3 and 1.3 for WCW). While furlough gave some job and financial protection, the lowest waged were least likely to have their pay topped up. Dropping to 80% of an already low wage brought financial risks to workers and WCW were by far the lowest waged.

**TABLE 4:** Summary binary logistic regression (full) models for furloughed and keyworkers.

	Furlough^b^		Key worker^c^									
	November 2020		April 2020		May 2020		June 2020		January 2021		March 2021	
	Odds ratio	95% CI	Odds ratio	95% CI	Odds ratio	95% CI	Odds ratio	95% CI	Odds ratio	95% CI		
Class and gender^a^												
Women ‐ working class	1.3**	(1.1, 1.6)	4.7***	(3.9, 5.7)	3.8***	(3.1, 4.7)	4.1***	(3.3, 5.1)	4.4***	(3.6, 5.5)	3.6***	(3.0, 4.3)
Women ‐ non working class	0.9	(0.8, 1.1)	1.8***	(1.5, 2.0)	1.7***	(1,4. 1.9)	1.9***	(1.7, 2.2)	1.7***	(1.5, 2.0)	1.8***	(1.6, 2.0)
Men ‐ working class	2.3***	(1.9, 2.9)	3.0***	(2.5, 3.6)	2.3***	(1.8, 2.8)	2.7***	(2.2, 3.4)	1.4***	(1.2, 1.8)	1.4***	(1.2, 1.8)
Constant	0.3***		0.8***		0.9*		0.87		0.79**		0.96	
Nagelkerke R square	0.09		0.28		0.28		0.29		0.11		0.09	
N	4953		7029		5966		5864		4830		5253	

*Note*: Employed women and men aged 18–65, excluding the self‐employed. Employed in month of focus and Wave j.

***p < 0.01, **p < 0.05, *p < 0.1.

*Source*: UKHLS weighted data.

^a^ Reference: Men non‐working class.

^b^ Furloughed in any previous month. Controls include current hours worked, age, ethnicity, couple, parental status.

^c^ Key workers. Controls include current hours worked, furlough (April/May/June), age, ethnicity, couple, parental status.

#### 5.2.3 Key workers

Finally, official lists were compiled of occupations deemed ‘key’ so that those workers were allowed to (and many had to) go out to work during lockdowns. This categorisation saw serious questions being asked in many countries about what roles and which workers are essential for societal functioning (Farris & Bergfeld, 2022; Farris et al., 2024; ILO, 2023; Remery et al., 2022). In recognising the critical need for certain types of work, being categorised as key was beneficial in protecting the workers from the worst threats to their jobs, but potentially put them at heightened risk of contact with the virus.

Working‐class women stood out as key workers each pandemic month. In logistic regressions, with added controls, they had significant odds ratios of 3.6 or higher (Table 4). Not only were WCW key, we know that they were also likely to be employed in face‐to‐face roles (e.g., in food and other necessary goods), jobs with high levels of social interaction and high risks of virus exposure. As Raphael and Schneider (2023) discuss, many WCW were newly contending with heightened emotional, contentious, and often dangerous interactions with clients and customers in their workplaces. Compared with non WCM, WCW stand out each wave, shaped by their higher representation in education and childcare, and health and social care sectors (the majority of male key workers were employed in transport. ONS, 2020).

## 6 DISCUSSION

The article explores the work of WCW in turbulent times. It focuses on the UK just prior to and during the Covid‐19 pandemic. The women's work, and the key skills involved, are fundamental to everyday lives, but both have been under‐valued and under‐rewarded. The pandemic shone a fresh light on the societal importance of the work of WCW, threw its undervaluation into stark relief, and revealed far‐reaching implications that go far beyond negative consequences for the individual worker and their families. Outcomes of the systemic low pay and difficult working conditions that WCW routinely face are labour shortages in key services, high turnover among pressurised staff, and a resulting inadequate provision that negatively impacts service users and their families as well as remaining key workers (Eurofound, 2023; Farris et al., 2024; ILO, 2023).

The article shows inequalities in paid work that WCW experienced in an analysis of nationally representative samples of thousands of workers. It makes three main contributions to the literature. The first is its case for recentring WCW in the sociology of work. We build upon 1980s feminist and class‐aware studies that, amid deep recessionary turmoil, showcased their working lives and both highlighted the importance of the work that WCW women do and demonstrated the necessity of a classed and gendered sociological analysis for identifying the wider effects of turmoil on working lives (Bradley, 1989; Cavendish, 1982; Crompton & Jones, 1984; Elson & Pearson, 1980; Pollert, 1981; Westwood, 1985). Attention to WCW's work has been more scattered across sociology since then, and class and gender have not necessarily been connected to help frame analyses of the women's work (MIlkman, et al., 2021). This article pulls together insights from the dedicated study of women's work, gender and class; from class‐focused non employment‐based research; and employment‐based non class‐focused analyses to argue that centring WCW's experiences of paid work remains a powerful tool for the sociology of inequalities, work and employment. As in the 1980s, centring WCW's paid work in this article reveals the wider ramifications of the pandemic for workers that include but reach beyond job loss, and the deep inequalities WCW were facing when compared with middle‐class workers and WCM.

The article's second contribution is thus to link literatures on the pandemic and on work lives amid turbulence more broadly. Covid‐19 has been illuminating for the sociology of work: working lives during the exceptional instability it triggered were highly unequal, but classed and gendered analyses have been rare (Chatot et al., 2023). Exploring patterns of employment with data gathered from thousands of workers, and just preceding and during the pandemic, we reveal the extent of disadvantage in the UK workplace as the pandemic arrived and as it shifted. The article provides up‐to‐date knowledge of WCW's paid work and finances, and stability and change therein, showing that the women's pre‐pandemic work‐time patterns and weak earnings persisted into 2020 and beyond. What also continued into the pandemic but grew in importance was WCW's lesser access to quality flexible working options to help manage (now intensified) work‐live pressures. Alongside continuities amid turbulence, new with the pandemic, WCW experienced more job loss than other workers, cuts in paid hours, stressful key working roles, alongside having to go out to work in unsafe environments, despite a key worker rhetoric that appeared to recognise their work. Key work was heavily relied upon in the crisis context, in contrast to pre‐pandemic years when many workers and much of their work were dismissed as low‐value and low skilled. Hales and Tyler (2022) argued that new recognition of the value of heroic workers was not linked to a commitment to better promote workplace equality and safe working conditions. Similarly here, the key worker rhetoric was not matched with proper recognition in the form of living wages, living hours, good working conditions and job security, impacting WCW disproportionately.

The third contribution of the article is to highlight the necessity for ongoing intersectional analyses of worlds of work that recognise when class, gender or both are the most appropriate explanations for inequalities old and new. In recentring WCW, comparing their work with WCM and with their middle‐class male and female peers, we show that some features of the women's employment were largely attributable to class, with some classed inequalities constant and others new with Covid‐19. New, and distinctly classed, for example, were such initiatives as the furlough scheme that saw large numbers of primarily working‐class workers doing no paid work – and taking a cut in wages ‐ while retaining their jobs. Meanwhile, in a covid‐led intensification of pre‐existing inequalities, very large numbers of primarily middle‐class workers worked fully from home and/or flexibly in other ways during extended periods of lockdown, new protections that were far less available to working‐class workers. As Dobusch and Kreissl (2020, p. 1) argue, the governance of workplace im/mobilities during the pandemic separated out ‘bodies perceived as highly valuable and worth protecting and those categorized as less valued and potentially disposable’: we demonstrate this was a firmly classed governance. We show clearly how different working‐ and middle‐class employment was prior to and amid the pandemic context but that gender also plays a crucial part. Before and during the pandemic, women's overall worktime and wages differed to those of men. With the pandemic, female employees, both working‐ and middle‐class, were more likely to be key workers than were male. However, we need the approach adopted in this article, exploring class and gender, to see that working‐class female workers were more likely to be key and employed in face‐to‐face roles than were middle‐class women, facing heightened risks.

In centering WCW and tracking thousands of workers, our research provides valuable insights into deep inequalities in working lives amid pandemic turmoil. The data afford an important overview of the key features of employment in the contemporary UK, allowing our article to describe important associations and patterns that disclose new possibilities for further research (Savage, 2024). In our focus on paid workers, for example, we did not explore those who lost jobs or were looking for employment amid the intense pressures of a global pandemic. Nor have we had space to examine important differences among WCW, including resulting from their specific occupational locations or due to such intersecting social divisions as age, ethnic group, migrant status, disability and more (Denis, 2008; Misra et al., 2021), all valuable possibilities for further research. Lastly, our article focuses on the period just prior to Covid‐19 and examines workers each month during it. Yet working lives were already in flux prior to the pandemic's arrival. A post‐recessionary decade was just coming to an end, marked by austerity politics, financial uncertainties, and growing precariousness in work, deepening due to the 2016 ‘Brexit’ vote and the official withdrawal from the EU in January 2020 as the pandemic loomed (Karamessini & Rubery, 2013; Rubery et al., 2024; Rubery & Rafferty, 2013). This period followed decades of growing instability and insecurity at work, declining job quality, especially in working‐class jobs alongside weakening collective worker voice (Henly et al., 2021).

Viewed positively, very unsettled times might open up opportunities for reversing these trends and equalizing inequalities (Elson, 2013; Hudde et al., 2023). Among uncertain times, the pandemic was noteworthy in that it created rapid initiatives, new worker protections and an urgency around defining whose work is valued in society, all amid hopes for a future better ‘new normal’ of work that calls out for further research in the post‐pandemic period (Beck et al., 2020). Continuing to recognise the types of work carried out by WCW can help advance the contemporary sociology of work and employment in this endeavour. Centring WCW in this article evidenced the unequal impacts of pandemic‐related turmoil on workers. It made clear that economic uncertainty has deep, wide and unequal ramifications that directly relate to class and gender. By expanding considerations of gendered and classed labour, contemporary research in sociology can generate more comprehensive and precise findings that better guide policy interventions designed to protect workers and reduce inequalities sharpened by turbulent times.

## CONFLICT OF INTEREST STATEMENT

The authors declare no conflict of interest.

## Supporting information

Supporting Information S1

## Data Availability

Our code is available from https://github.com/luistorresr/gender_covid_uk.
