# Supplementary material for: Class, gender and the work of working‐class women amid turbulent times
Source: Br J Sociol. 2024 Sep 19;76(1):96–113. doi: 10.1111/1468-4446.13147 (PMC11717167; doi:10.1111/1468-4446.13147)
Supplement: Supplementary file 1 — Supporting Information S1 [file BJOS-76-96-s001.pdf]

Appendix 1. Sample characteristics

a. Labour Force Survey

Year	Gender	Category	Jan/Mar	Apr/Jun	Jul/Sep	Oct/Dec
2019	Men	Management/professional	6,728,261	6,731,414	6,863,603	6,907,353
		Intermediate	1,208,658	1,245,184	1,205,814	1,266,052
		Lower supervisory/technical	1,699,311	1,712,139	1,741,468	1,704,750
		Routine/semi-routine	3,364,106	3,292,744	3,295,891	3,275,407
		**Total**	13,000,336	12,981,481	13,106,776	13,153,562
	Women	Management/professional	6,113,554	6,131,552	6,183,442	6,336,911
		Intermediate	2,832,868	2,885,006	2,880,074	2,771,328
		Lower supervisory/technical	602,949	591,889	588,869	601,581
		Routine/semi-routine	3,213,068	3,167,516	3,154,762	3,147,656
		**Total**	12,762,439	12,775,963	12,807,147	12,857,476
2020	Men	Management/professional	6,905,995	6,953,228	7,172,913	7,209,423
		Intermediate	1,313,323	1,373,499	1,365,175	1,476,236
		Lower supervisory/technical	1,701,316	1,695,255	1,599,954	1,492,292
		Routine/semi-routine	3,184,360	3,145,097	3,039,494	2,979,188
		**Total**	13,104,994	13,167,079	13,177,536	13,157,139
	Women	Management/professional	6,332,061	6,387,886	6,456,446	6,557,810
		Intermediate	2,746,174	2,777,923	2,835,161	2,917,818
		Lower supervisory/technical	602,663	596,680	598,551	559,629
		Routine/semi-routine	3,168,075	3,030,194	2,967,824	2,867,043
		**Total**	12,848,973	12,792,683	12,857,982	12,902,300
2021	Men	Management/professional	7,264,709	7,256,871	7,326,901	7,353,247
		Intermediate	1,443,975	1,397,544	1,458,052	1,465,523
		Lower supervisory/technical	1,370,735	1,298,471	1,246,984	1,266,534
		Routine/semi-routine	3,103,273	3,153,698	3,234,614	3,215,024
		**Total**	13,182,692	13,106,584	13,266,551	13,300,328
	Women	Management/professional	6,609,478	6,506,232	6,554,774	6,688,168
		Intermediate	2,961,105	2,999,907	3,029,344	2,958,302
		Lower supervisory/technical	428,920	438,044	458,720	447,549
		Routine/semi-routine	2,877,698	2,898,073	2,894,581	2,863,998
		**Total**	12,877,201	12,842,256	12,937,419	12,958,017

b. the UK Household Longitudinal Study

		**2018-19**	**2020**						**2021**		
			**April**	**May**	**June**	**July**	**Sept**	**Nov**	**Jan**	**March**	**Sept**
Men	Management/professional	3,652	1,767	1,578	1,493	1,422	1,357	1,271	1,234	1,346	1,360
	Intermediate	752	407	348	315	319	276	229	259	293	285
	Lower supervisory/technical	776	441	408	372	378	343	286	329	353	316
	Routine/semi-routine	1,934	845	762	702	738	617	614	601	569	623
	**Total**	**7,114**	**3,460**	**3,096**	**2,882**	**2,857**	**2,593**	**2,400**	**2,423**	**2,561**	**2,584**
Women	Management/professional	3,533	1,795	1,604	1,535	1,503	1,415	1,338	1,307	1,359	1,409
	Intermediate	1,446	768	685	647	594	587	556	512	576	589
	Lower supervisory/technical	330	168	168	143	147	125	133	130	119	122
	Routine/semi-routine	2,378	1,183	1,051	1,008	968	912	826	841	889	892
	**Total**	**7,687**	**3,914**	**3,508**	**3,333**	**3,212**	**3,039**	**2,853**	**2,790**	**2,943**	**3,012**

Note:
In employment aged 18-65, excluding the self-employed (weighted each wave using cross-sectional weights).

Appendix 2. List of variables

Category	Variable name	Survey	Description	Categories range
Respondent characteristics	SEX	LFS / UKHLS	Sex of respondent	Male / Female
	AGE		Age of respondent	Numeric value
Jobs	NSECMJ10<br>NSECMJ20 (update)<br>NS-SEC	LFS<br>LFS<br>UKHLS	National Statistics Socio-Economic Classification major group	Management & professional; Intermediate; Small employers & own account; Lower supervisory & technical; Semi-routine and routine
	ILODEFR	LFS	Basic economic activity (ILO definition) (reported)	In employment, ILO unemployed, Inactive, Under 16
	INECAC05	LFS	Basic economic activity (ILO definition) (reported)	34 categories including employee, self-employee and unemployed
	STAT<br>STATR	LFS	Employment status and employment status in main job (reported)	Employee; Self-employed; Government scheme; Unpaid family worker
	Keyworker status	UKHLS	Are you working as a key worker in any of the key sectors below during the current coronavirus situation?<br>1. Health and social care<br>2. Education and childcare<br>3. Key public services<br>4. Local and national government<br>5. Food and other necessary goods<br>6. Public safety and national security<br>7. Transport<br>8. Utilities, communications and financial services<br>9. No, I am not working as a key worker	Yes<br>No<br>I am not working as a key worker
	PUBLICR	LFS	Public or private sector (reported)	Private / Public
Flexible work arrangements (FWA)	FLED10	LFS	Type of agreed work arrangement	Flexitime; Annualised hours contract; Term time working; Job sharing; Nine-day fortnight; Four-and-a-half-day week; Zero hours contract; On-Call Working; None of these; Don’t know
	FTPTWK	LFS	Whether full or part time in main job	Full-time; Part-time
	Formal FWAs access	UKHLS	If you personally needed any, which of the following arrangements for flexible working are available at your workplace? (Select all that apply).	Part-time working; Term-time only; Job sharing; Flexitime; Compressed hours; Annualised Hours; Working from home on a regular basis; Zero-hours contract; On-call; Other; None of these
	Informal FWAs	UKHLS	Aside from any formal arrangements for flexible working you have, are you able to vary your working hours on an informal basis, for example by re-arranging your start or finish times if you need to?	Yes<br>No<br>Sometimes
	Working at home	UKHLS	During [the last four weeks/ January and February]: how often did you work at home?	Always<br>Often<br>Sometimes<br>Never
	HOME	LFS	Whether mainly work from home (main job)	In own home; In the same grounds or buildings as home; In different places using home as a

				base; Somewhere quite separate from home
Weekly hours worked	BACTHR	LFS	Basic actual hours in main job (per week)	(0-96) Number of hours; (97) 97 hours or more
	Current	UKHLS	How many hours did you work, as an employee or self-employed, last week? Please include all jobs and self-employment activities. If you didn't work any hours in your job(s), please enter zero	Number of hours
	Baseline	UKHLS	During January and February 2020, how many hours did you usually work per week? Please include all jobs and self-employment activities. If you didn't work any hours in your job(s), please enter zero.	Number of hours
Financial situations	Wages	UKHLS	What is your usual take-home pay/earnings now? Please include all jobs and self- employment activities.	In £s
	Managing financially	UKHLS	How well would you yourself say you are managing financially these days?	'Living comfortably' to 'Finding it very difficult'
